# Changes in the Pharmacokinetics and Pharmacodynamics of Sildenafil in Cigarette and Cannabis Smokers

**DOI:** 10.3390/pharmaceutics13060876

**Published:** 2021-06-13

**Authors:** Mohammed Murtadha, Mohamed Ahmed Raslan, Sarah Farid Fahmy, Nagwa Ali Sabri

**Affiliations:** 1 Department of Clinical Pharmacy, Ain Shams University, Cairo 11865, Egypt; mohamed.hussain18@pharma.asu.edu.eg (M.M.); mohamed.raslan@pharma.asu.edu.eg (M.A.R.); Sarah.farid@pharma.asu.edu.eg (S.F.F.); 2 Drug Research Center, Clinical Research and Bioanalysis Department, Cairo 11865, Egypt

**Keywords:** sildenafil, cannabis, smokers, pharmacokinetics, pharmacodynamics, erectile dysfunction, cytochrome P3A4, drug interactions, oxidative stress, safety

## Abstract

Sildenafil citrate, a widely-used oral therapy for erectile dysfunction, is a cytochrome P3A4 (CYP3A4) enzyme substrate. Studies have reported that this substrate has an inhibitory effect on CYP3A4 enzymes in long-term cigarette and cannabis smokers, which predominantly mediate the hepatic elimination of sildenafil. Cigarette and/or cannabis smoking could therefore alter the exposure of sildenafil. The aim of this study was to examine the effect of smoking cigarettes and/or cannabis on the pharmacokinetics, pharmacodynamics, safety and tolerability of sildenafil. Thirty-six healthy human subjects were equally divided into three groups: non-smokers, cigarette smokers and cannabis smokers. Each group was administered a single dose of sildenafil (50 mg tablets). The primary outcome measures included the maximum concentration of sildenafil in plasma (C_max_), the elimination half-life (t1/2) and the area under the plasma concentration time curve from zero to time (AUC_0–t_). The pharmacodynamics were assessed by the International Index of Erectile Function (IIEF-5). The exposure of sildenafil (AUC_0–t_) showed a statistically significant increase in cigarette smokers (1156 ± 542 ng·h/mL) of 61% (*p* < 0.05) while in cannabis smokers (967 ± 262 ng·h/mL), a non-significant increase in AUC_0–t_ of 35% (*p* > 0.05) was observed relative to non-smokers (717 ± 311 ng·h/mL). Moreover, the C_max_ of sildenafil increased by 63% (*p* < 0.05) and 22% (*p* > 0.05) in cigarette smokers and cannabis smokers, respectively. Cigarette smoking increases the exposure of sildenafil to a statistically significant level with no effect on its pharmacodynamics, safety and tolerability.

## 1. Introduction

Sildenafil citrate is a PDE-5 inhibitor that is administered orally, which has proved to be an effective therapy for male erectile dysfunction (ED) resulting from organic dysfunction, psychological origin or a mixed etiology. Moreover, sildenafil has been proven to be highly effective in clinical settings with a wide safety margin, patient satisfaction and improved quality of life in most patients [1,2,3].

The prevalence of erectile dysfunction (ED) increases with age and is expected to continue to rise globally, with approximately 322 million men affected by 2025 due to the increase in the number of elderly people worldwide. The availability of PDE-5 has resulted in an increase in the number of patients searching for medical assistance for erectile dysfunction and has substantially altered the medical treatments available for ED. More than thirty million men around the globe were being treated with sildenafil by 2006, a figure that has certainly increased since then [4].

The parameters of the pharmacokinetics following the administration of sildenafil exhibit rapid absorption with an absolute bioavailability of 40%. Sildenafil reaches a peak plasma concentration within 30 min to 2 h (median 60 min) under fasting conditions. Sildenafil is mainly metabolized by the cytochrome P450 hepatic isoenzymes 3A4 (a major pathway) and 2C9 (a minor route) [5]. CYP450 converts sildenafil to an active form of N-desmethyl metabolite, which has been shown to contribute to approximately half of the potency and activity of sildenafil. Sildenafil and the N-desmethyl metabolite are highly bound to plasma proteins (96%) and their terminal half-lives are 4 h [6]. It is worth mentioning that 75% or more of the N-demethylation of sildenafil is attributable to the effect of CYP3A4 on sildenafil metabolism [7].

Azole antifungal drugs, macrolide antibiotics, antiviral drugs such as ritonavir and grapefruit juice are proven CYP3A4 inhibitors. Upon a co-administration with sildenafil, they may cause the elevation and prolongation of its serum concentrations and, consequently, the enhancement of its pharmacological and toxicological effects [8,9].

A pharmacokinetic study was conducted on 28 subjects to investigate the interactions between sildenafil and ritonavir, a CYP3A4 inhibitor. After multiple drug doses, it was shown that ritonavir resulted in a remarkable increase in sildenafil The AUC increased 11-fold and the C_max_ by 3.9-fold and there was a marked delay in T_max_ by 3.1 h (*p* = 0.0018) relative to the placebo. The obtained results showed that both the safety and tolerability of sildenafil are highly altered, indicating a possible occurrence of adverse events unless the dose does not exceed 25 mg upon a concomitant administration with ritonavir [3].

A study of the effect of grapefruit juice on the pharmacokinetics of sildenafil found that grapefruit juice increased the AUC_0–inf_ of sildenafil by 23% and the T_max_ period was delayed by 0.25 h, suggesting that grapefruit juice enhances the rate and extent of the absorption of sildenafil; therefore, it is not advisable to consume grapefruit juice alongside sildenafil treatment [10].

Recently, a study showed the effects of genetic polymorphisms in CYP3A4 and CYP2C9 and their impact on sildenafil metabolism [11]. Additionally, a few studies were carried out in Egypt that showed how sildenafil metabolism could be affected by inter-individual variability in CYP3A4 and CYP2C9 in the Egyptian population [12,13].

Smoking is associated with an increased risk of ED, meaning that sildenafil is likely to be frequently administered by smokers [14]. In a preclinical study, long-term smoking was reported to have an inhibitory effect on CYP3A4, indicating that CYP3A4 is likely to be inhibited to some extent in smokers. As a result, smoking could alter the clearance and plasma concentrations of the CYP3A4 substrate drug, sildenafil [15].

Moreover, cannabis, a widely-abused herb, is known to inhibit the predominant route of sildenafil metabolism via the CYP3A4 pathway [16]. The possible alterations in the pharmacokinetics of sildenafil in cigarette and/or cannabis smokers might also negatively alter the safety profile of sildenafil. The United States (US) Food and Drug Administration (FDA) recently reported that significant cardiovascular events including sudden cardiac death have occurred in men with erectile dysfunction who took sildenafil citrate. These reports have raised concerns that sildenafil citrate might increase the risk of cardiovascular events, particularly fatal arrhythmias, in patients with cardiovascular disease [17,18].

The possibility that sildenafil may be co-administered with cannabis smoking is likely to have consequences on the cardiovascular system. Indeed, a published case report of a 41-year-old man with a myocardial infarction who co-administered sildenafil with cannabis found that the patient exhibited increased sildenafil plasma levels. Consequently, the physiologic and adverse effects were combined [19].

Investigations have indicated that nicotine, which is the main psychoactive compound in cigarettes, stimulates Interleukin-6 (IL-6) expression, proven by experimental work on cells treated with nicotine [20]. On the other hand, in vitro experimental studies showed that IL-6 cytokine release during inflammatory events is mainly responsible for CYP3A4 downregulation [21]. These facts lead to the suggestion that smoking induces IL-6 expression, which, in turn, downregulates CYP3A4, the primary enzyme responsible for sildenafil metabolism.

Therefore, the aim of this study was to examine the effect of cannabis and/or cigarette smoking on the pharmacokinetics, pharmacodynamics, safety and tolerability of sildenafil and to measure the total antioxidant capacity and malondialdehyde levels as oxidative stress parameters in smokers.

## 2. Methods

### 2.1. Design

**Figure 1:**
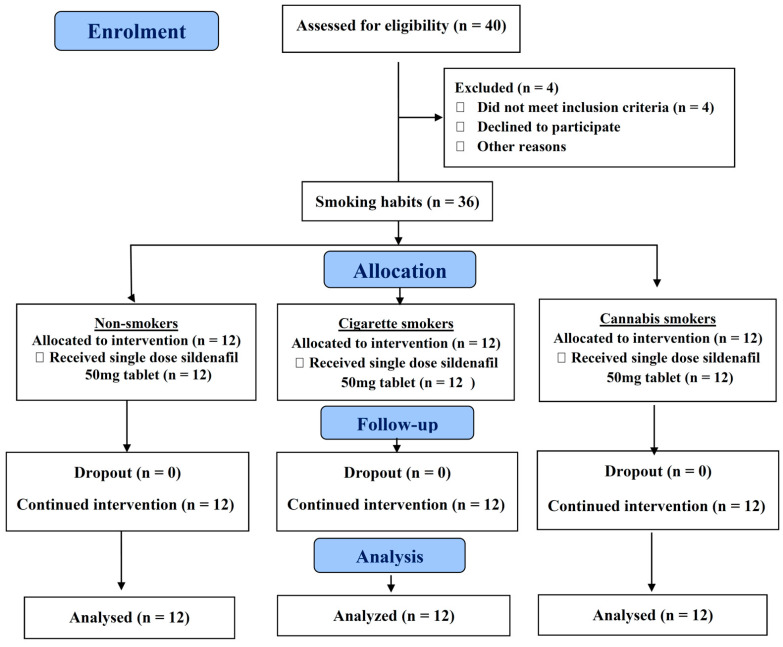
The study flow chart (consort diagram).

The study was a randomized open-label parallel study with the volunteers chosen randomly and then assigned according to their smoking habits. Thirty-six (*n* = 36) subjects were divided into three groups: Group 1 (*n* = 12) included non-smokers, Group 2 (*n* = 12) included cigarette smokers and Group 3 (*n* = 12) included cannabis smokers. Figure 1 shows the study flow chart.

### 2.2. Ethics

The study was conducted in accordance with the Declaration of Helsinki and the good clinical practice (GCP) guidelines adopted by the International Council for Harmonization (ICH).

This study was registered on ClinicalTrials.gov (Identifier: NCT04100759). The Drug Research Center ethics committee approved the study (Study Code: SIL-RES-BS-0119-0012, September 24, 2019) and the essential documents were all archived according to the Drug Research Center’s internal procedures for authorized direct access.

The Ethical Committee of the Faculty of Pharmacy, Ain Shams University, also approved the study (approval number: 222, December 15, 2018). All participants gave their informed consent.

The clinical investigator, study director (principal investigator) and licensed physicians were responsible for the physical examination and follow-up of the subjects for the appearance of any side effects or adverse events and the evaluation of vital signs throughout the study.

### 2.3. Subjects

All subjects were subjected to a screening examination prior to enrolment in the study. The medical history, concomitant medications, physical examination results, clinical laboratory tests and blood pressure measurements were recorded for each subject.

#### 2.3.1. Inclusion Criteria

Volunteers were between 18 to 55 years old with a calculated body mass index (BMI) within the normal acceptable limits, showing no history of contribution to any pharmacokinetics study within the past three months before the study date, a normal physical examination and laboratory data within normal limits. The subjects were not alcoholics or drug abusers and were not known to have any history of either. The study included non-smokers and cannabis smokers (frequent smokers) with an average smoking habit of 1–2 times per week within the past 70 days prior to the study as well as cigarette smokers (everyday smokers) with an average smoking habit of 5–10 cigarettes/day for at least five years.

#### 2.3.2. Exclusion Criteria

The exclusion criteria were a known drug or excipient hypersensitivity, gastrointestinal tract disorders, autoimmune diseases, renal diseases or renal dysfunction. Moreover, the following exclusions were also made: cardiovascular diseases, diabetes, hepatobiliary disease, hematological abnormalities, respiratory diseases, neurological diseases, patients with endocrine disease, excessive alcohol intake or drug abuse history, those who were positive for HIV-I, had abnormal laboratory values, any prior surgery of the gastrointestinal tract that might interfere with the drug absorption, treatment with any known hepatic enzyme-inducing/inhibiting agents within 30 days prior to the start of the study and throughout the study, treatment with any known CYP3A4 inhibitor or inducer drug, those who had been administered any medication less than two weeks before the study start date and any subjects who had donated blood or who participated in clinical studies that required multiple blood sample collections within the 1.5 months prior to the study start date.

### 2.4. Bioanalytical Method

A simple liquid-liquid extraction (LLE) method was used for the extraction of sildenafil from plasma by the addition of 3 mL of extraction solvent (7 diethyl ether: 3 dichloromethane *v*/*v*), followed by the alkalinization of 500 μL of plasma with 100 μL of 0.25 N sodium hydroxide (NaOH) and the addition of 50 μL of vardenafil (internal standard) (700 ng/mL). After vortex mixing for 2 min, samples treated by LLE were centrifuged at 4000 rpm for 5 min (Hermle Z 326 K, Hermle Labortechnik GmbH, Wehingen, Germany) followed by the separation of the organic layer (~2.5 mL). The organic extract was evaporated to dryness in a concentrator at 45 °C (Vacufuge^®^ Plus, Eppendorf, Germany). The resulting dry residue from the concentration process was reconstituted in 200 μL of the mobile phase and vortex mixed for 1 min. A sum of 2.5 μL was then injected for the purpose of the LC-MS/MS analysis, as shown in Table S1 (Bioanalytical Supplementary Materials).

The plasma level of sildenafil was determined using high-performance liquid chromatography-tandem mass spectrometry (Agilent Technologies, Inc., Santa Clara, CA, USA). The chromatographic system (LC Agilent 1200 series, Agilent Technologies, Santa Clara, CA, USA) consisted of a mobile phase of 25 mM ammonium acetate: methanol 25:75 *v*/*v* at a flow rate of 0.7 mL/min. The elution was performed using a C_18_ analytical column (Phenomenex Luna C_18_ 50 × 4.6 mm p.s. 5 µm).

The mass detector (Agilent 6410 triple quadrupole mass spectrometer, Agilent Technologies, Santa Clara, CA, USA) was operated in positive electrospray ionization mode (ESI). Multiple reaction monitoring modes (MRM) were used to detect precursor ion transitions to the production of sildenafil (m/z = 475 → 100). Mass Hunter software (Mass Hunter Workstation Software; Quantitative Analysis version B.04, Agilent Technologies, Inc. Santa Clara, CA, USA), using the same processing parameters such as integration type, processed all of the chromatograms in the same batch automatically, as shown in Table S3 (Bioanalytical Supplementary Materials).

Malondialdehyde (MDA) was determined by a reaction with thiobarbituric acid (TBA) in an acidic medium at a temperature of 95 °C for 1 h, producing a colored product. The pink reaction product was measured at 534 nm [22].

### 2.5. Safety and Tolerability

The participants were interviewed and data were collected with regard to their medical and medication history and a complete physical examination and clinical laboratory investigations were carried out.

The reporting of the incidence of adverse effects and/or side effects was performed during the study phases. In addition to monitoring adverse events and compliance throughout the study, the characterization of the safety and tolerability profiles was employed by monitoring the blood pressure and pulse rate at different time intervals post-dose. The recorded changes in baseline (pre-dose) blood pressure and pulse rate were co-plotted with the plasma concentrations of sildenafil at the same time intervals for assessment.

- Dose administration:

Subjects were admitted for residency in a clinical setting one day before the dose administration for control purposes. After an overnight fasting period of 8–10 h, the subjects in each of the three groups were administered a single oral dose of Viagra^®^ 50 mg film-coated tablets (50 mg of sildenafil) ingested with 240 mL of water. For compliance control, on the ambulant dosing study day, subjects were under close surveillance by qualified staff throughout the whole period of the study. Two different licensed nurses checked each subject for the complete ingestion of the dose using a wooden tongue depressor. The diet of the subjects in this study is available in Pharmacokinetic Supplementary Materials Table S12. The total number of calories given was 850 for breakfast and 1090 for lunch. Additionally, all volunteers in the smokers group smoked one cigarette before the administration of a single oral dose of sildenafil and 2 h after the dose (up to a period of 12 h), the smokers smoked another cigarette.

- Sample collection:

Urine samples were collected in coded cups for multi-drug screening tests, which were then used to recall participants for recruitment in our study after qualification and the application of the inclusion and exclusion criteria. It is worth mentioning that, in this study, subjects were re-screened using multi-drug screen test cards prior to the dose administration to qualitatively test for THC and commonly-abused drugs in urine samples in order to avoid a possible confounding effect from other drugs. Subjects with a positive THC multi-drug screen test and negative results for any other tested commonly-abused drugs were included in the cannabis smokers’ group if they fulfilled the other required criteria. The number of required blood samples and their disposition after collection were designed according to the pharmacokinetics of sildenafil. After the cannulation of the subjects’ forearm vein, blood samples were collected in tubes containing ethylene-diamine-tetra acetic acid (EDTA) at the following intervals: pre-dose, 10, 20, 30 and 45 min and 1, 1.5, 2, 3, 4, 6, 8, 10, 12 and 24 h after the drug administration. The collected blood samples were centrifuged at 5000 rpm for 5 min to separate the plasma. The collected plasma was stored at −80°C until the sample analysis.

- Pharmacokinetics analysis:

The pharmacokinetics of sildenafil were characterized by a non-compartmental analysis (WinNonlin version 2.0, Pharsight, California, Palo Alto, CA, USA). We determined the peak concentration of sildenafil in plasma C_max_ and the area under the plasma concentration time curve from zero to time (AUC_0–t_) as the primary pharmacokinetic parameters. The time to peak plasma concentration (T_max_), elimination half-life in plasma (t_1⁄2_) and the area under the plasma concentration time curve from zero to infinity (AUC_0–inf_) were calculated as secondary parameters. The terminal log-linear part of each plasma concentration time curve was identified by an automatic function integrated into the software in order to calculate the elimination rate constant k_el_ (h^−1^) by a linear regression analysis. The t_1⁄2_ (h) was calculated automatically by the software using the following equation: t_1⁄2_ = 0.693/K_el_. The AUC values were also calculated automatically by the linear trapezoidal method.

- Pharmacodynamics analysis:

The pharmacodynamic response of sildenafil was assessed subjectively by the international index of erectile function (IIEF-5) [23]. Malondialdehyde (MDA) and the total antioxidant capacity levels were determined as the surrogate biomarkers for oxidative stress. Oxidative stress is susceptible to elevation in smokers. Furthermore, MDA plasma levels reflect the degree of lipid peroxidation.

- Statistical analysis:

The pharmacokinetic parameters were calculated and the results presented as the mean ± standard deviation (SD). Statistical comparisons were made by a one-way analysis of variance (ANOVA) of the AUC_0–∞_ and C_max_. All statistical analyses were performed using SAS software (SAS Institute Inc., Cary, NC, USA). A *p*-value less than 0.05 was considered statistically significant and was calculated automatically by the linear trapezoidal method.

## 3. Results

### 3.1. Subjects

No dropouts occurred throughout the study period. All subjects showed a normal clinical profile upon physical assessment and clinical and biochemical laboratory testing. A demographic data analysis revealed no significant differences in age and body mass index among the study groups, as shown in Table 1.

### 3.2. Bioanalytical Results

The liquid chromatography-tandem mass spectrometric method was validated following the US FDA Bioanalytical Method Validation Guidance validation parameters [24]. The calibration curves were reliable, reproducible and linear for a concentration range of 1–500 ng/mL with a regression coefficient (r^2^) = 0.999. The accuracy and precision of the quality control samples were adequate and within the acceptable limits of ≤ 20% for the lower limit of quantitation (LLOQ) and ≤ 15% for the three levels of low, medium and high concentrations of the quality control samples. The ranges of percent accuracy and precision were 88–101% and 92–102%, respectively, as shown in Table 2.

### 3.3. Pharmacokinetics and Statistical Analysis

**Figure 2:**
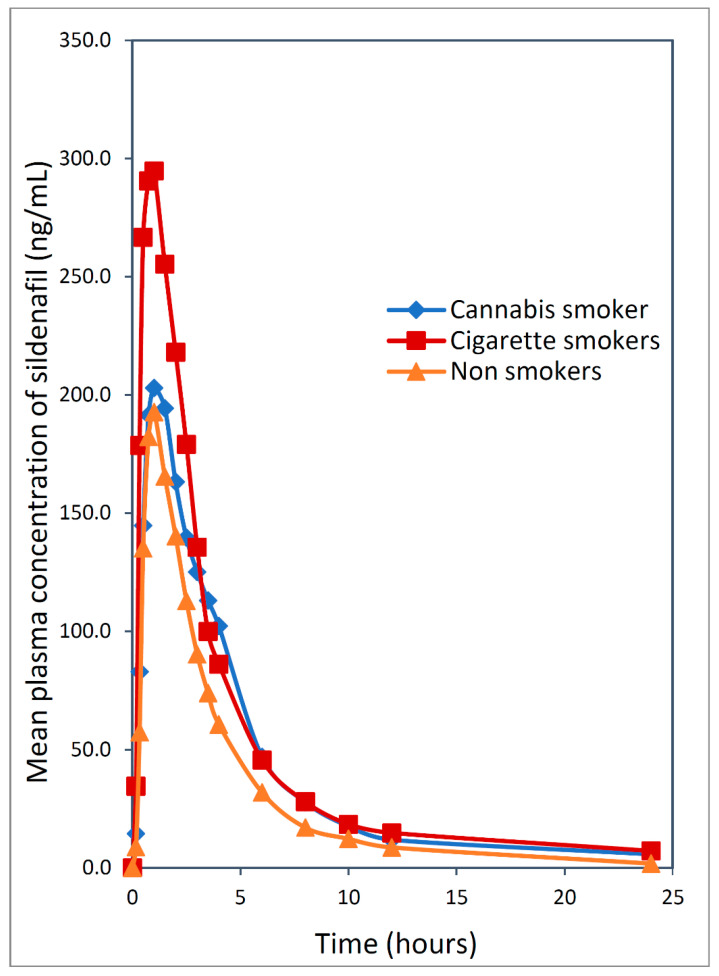
Mean sildenafil plasma concentration versus time curves after the oral administration of a single 50 mg dose of sildenafil in each group.

Compared with the non-smokers’ group, the exposure of sildenafil (AUC_0–t_) significantly increased in cigarette smokers by 61% (*p* < 0.05) while in cannabis smokers, a non-significant increase in the AUC_0–t_ of 35% (*p* > 0.05) was observed. Moreover, the C_max_ of sildenafil increased by 63% (*p* < 0.05) and 22% (*p* > 0.05) in cigarette smokers and cannabis smokers, respectively, as shown in Table 3 and Figure 2.

The least significant difference pairwise comparison showed a significant difference in the AUC_0–t_ between cigarette smokers and non-smokers (*p* = 0.03) with no significant differences between cannabis smokers and cigarette smokers or non-smokers (*p* > 0.05). Regarding the AUC_0–inf_, there was a significant difference between cigarette smokers and non-smokers while no significant difference was found between cannabis smokers and either cigarette smokers or non-smokers. The T_max_ was comparable among the three groups of the study.

### 3.4. Safety, Tolerability and Pharmacodynamics

Sildenafil was well tolerated throughout the study despite the mild adverse events experienced by a few of the subjects. No subjects withdrew from the study for any reason attributable to the drug and no laboratory or vital sign abnormalities were observed.

The numbers of incidents of adverse events in each group (*n* = 12) were as follows: non-smokers (*n* = 5), smokers (*n* = 4) and cannabis smokers (*n* = 3). Adverse events were mild and resolved quickly. A headache was the most common adverse event in all groups. One subject in the cannabis group had a headache with flushing.

**Figure 3:**
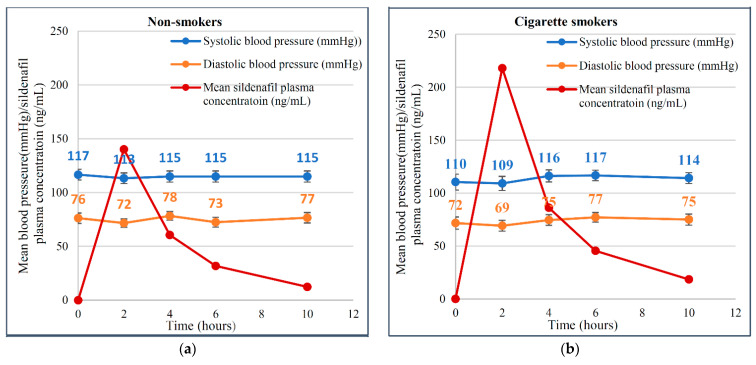

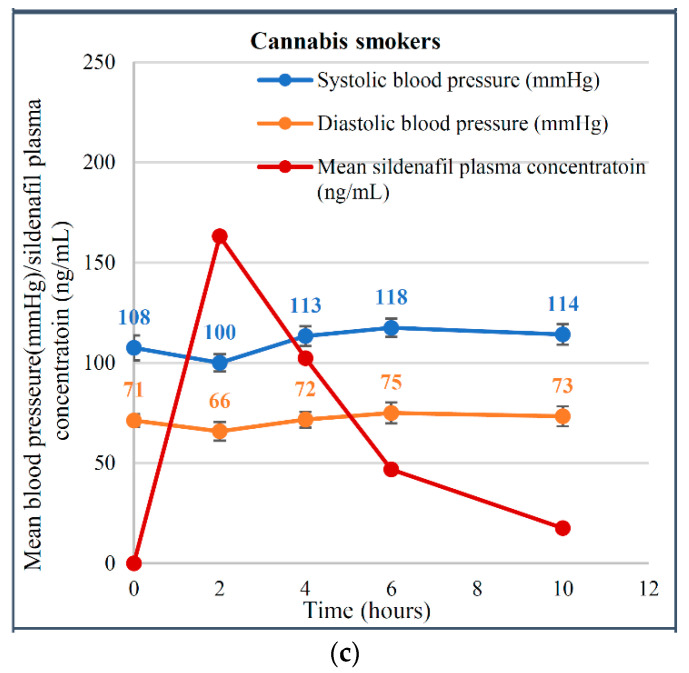
Mean blood pressure (systolic/diastolic) co-plot with mean sildenafil plasma concentration versus time after the oral administration of a single 50 mg dose of sildenafil tablets in each group: (**a**) non-smokers; (**b**) cigarette smokers; (**c**) cannabis smokers.

**Figure 4:**
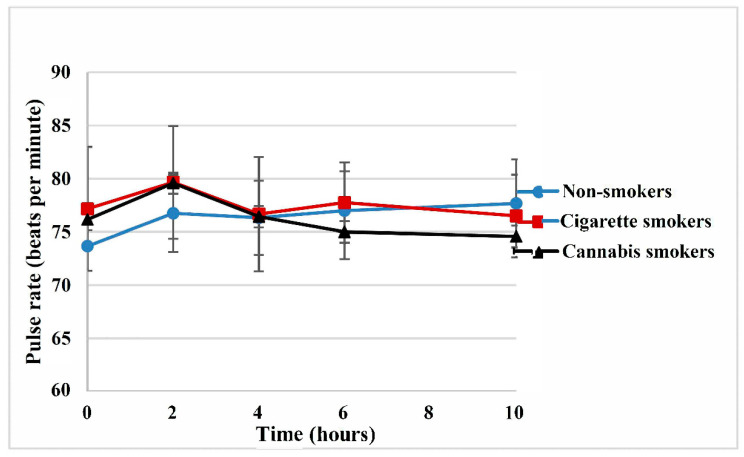
Mean pulse rate after a single oral dose of sildenafil 50 mg tablets in all study groups.

At the zero-time point (pre-dose) and after 10 h post-dose, the mean systolic blood pressure values were 117 and 115 mmHg for non-smokers, 110 and 114 mmHg for cigarette smokers and 108 and 114 mmHg for cannabis smokers, respectively. The diastolic blood pressure at zero-time (pre-dose) and after 10 h post-dose were 76 and 77 mmHg for non-smokers, 72 and 75 mmHg for cigarette smokers and 71 and 73 mmHg for cannabis smokers, respectively. At two-hour intervals post-dose, the mean systolic blood pressure values were 113, 109 and 100 mmHg for non-smokers, cigarette smokers and cannabis smokers, respectively. The diastolic blood pressure values at two hours were 72, 69 and 66 mmHg for non-smokers, cigarette smokers and cannabis smokers, respectively. There were no clinically significant changes in blood pressure or pulse rate among the three groups of the study. However, the decrease in blood pressure was most notable at the 2 h interval post-dose, corresponding with sildenafil plasma concentrations of 140, 218 and 163 ng/mL in non-smokers, cigarette smokers and cannabis smokers, respectively, as shown in Figure 3. The pulse rates were comparable among the three groups with average pulse rates of 76, 78 and 76 beats per minute over a 10 h interval post-dose in non-smokers, cigarette smokers and cannabis smokers, respectively, as shown in Figure 4 and Tables S15–S17 (Supplementary Materials).

The one-way ANOVA showed a statistical significance for the difference in the total antioxidant capacity (TAC) among the study groups. The pairwise comparison showed a significant difference in the TAC in cigarette smokers and cannabis smokers, which was relevant to the non-smokers’ group (*p* < 0.05). The MDA concentrations were not significantly different between each of the study groups (*p* > 0.05), as shown in Table 4. The IIEF-5 scores were not significantly different between the three groups, with scores of 23, 21 and 21 for non-smokers, cigarette smokers and cannabis smokers, respectively.

## 4. Discussion

The pharmacodynamic and pharmacokinetic profiles of sildenafil are important contributing factors to its effectiveness in treating men with erectile dysfunction of various etiologies [25]. A recent study reported a strong association between males smoking marijuana and taking unprescribed sildenafil tablets, which might evoke drug-drug interactions. As a result, the potential for drug interactions and their associated risks with the concomitant use of marijuana and PDE-5 inhibitors should be studied [26].

It is important to mention that the potential interaction of cannabis (perpetrator) with sildenafil (victim drug) is of particular interest as it is well known that cannabis is an inhibitor of the hepatic CYP450 isoform 3A4 [27,28], which is the major isoform that metabolizes sildenafil. In the current study, following a single oral dose of sildenafil in each of the different groups, there was a significant increase in the AUC_0–∞_, C_max_ and AUC_0–t_ in the cigarette smokers compared with the non-smokers while no significant difference was detected when comparing cannabis smokers with non-smokers. Cigarette smoking can affect drug metabolism through different pharmacokinetic and pharmacodynamic mechanisms and a change in smoking habits can leave patients at a high risk of serious adverse reactions [29]. In the current study, smoking significantly increased the sildenafil bioavailability and its maximum plasma concentration (C_max_) in cigarette smokers relative to non-smokers. CYP2C9 may be involved in the increased exposure of sildenafil in smokers [30].

On the other hand, the CYP3A4-mediated metabolism of sildenafil is the major metabolic pathway of sildenafil. The effect of cigarette smoking on CYP3A4 is not extensively studied and represents a controversial subject [31]. Long-term smoking is reported to have an inhibitory effect on CYP3A4, indicating that CYP3A4 is likely to be inhibited to some extent in smokers. As a result, smoking could possibly alter the clearance and plasma concentrations of the CYP3A4 substrate drug, sildenafil [15].

After the administration of 50 mg of sildenafil to healthy subjects, Nichols et al. reported an AUC_0–t_ of 727 ng·h/mL, similar to the AUC_0–t_ of 717.4 ng·h/mL that we achieved for the same dose of sildenafil in this study’s healthy non-smokers’ group. Interestingly, cigarette smokers had an AUC_0–t_ of 1155.9 ng·h/mL, approaching an approximate AUC_0–t_ of 1667 ng·h/mL after the administration of 100 mg of sildenafil in the study by Nichols et al. [25]. Sildenafil is a lipophilic compound with a pH-dependent solubility that is highly enhanced at pH 1.2 and 4.5 [32]. In addition to the inhibitory effect of cigarette smoking on CYP3A4, smoking is associated with an increase in gastric acid secretion and maintains acidity throughout the gastrointestinal tract (GIT) by reducing the secretion of bicarbonate, a crucial neutralizer for acidity in the duodenum [33,34]. In addition, smoking causes an increase in the gastric emptying rate [35]. Therefore, smoking could be associated with the enhanced solubility and dissolution of sildenafil throughout the GIT, consequently increasing the extent of the absorption of sildenafil [33]. Unlike cigarette smoking, cannabis causes a delay in the gastric emptying rates and a reduction in gastric acid secretion, which might have outweighed its inhibitory effect on CYP3A4 in the current study and resulted in a lesser enhancement of the exposure of sildenafil than cigarette smoking [35,36]. Moreover, the non-significant effect of cannabis on the exposure of sildenafil that we observed might also be correlated with our single dose study design. In addition, the uncontrolled delivery of cannabis in cigarette smoke predisposes patients to inter-subject variations in exposure.

The pharmacokinetic alteration of sildenafil exposure in cigarette smokers was probably due to the effect of smoking on the hepatic metabolism of sildenafil, which is mediated by liver cytochrome enzymes (CYP3A4) or the effects of genetic polymorphisms in CYP3A4 and CYP2C9 and their impact on sildenafil metabolism, which is subject to inter-individual variability in CYP3A4 and CYP2C9 in the Egyptian population [12,13].

Smoking is one of the main causes of oxidative stress, producing various free radicals in the body and ultimately causing damage to vital macromolecules [37,38]. The total antioxidant capacity (TAC) and plasma malondialdehyde (MDA) have been considered important surrogate biomarkers of oxidative stress evaluation [39,40]. A lower TAC was observed in the present study in cigarette smokers relative to non-smokers (as in Table 4). This result is in agreement with the results obtained by Ranjbar et al., who reported a reduction in the TAC in smokers relative to non-smokers [41]. Moreover, smoking was also associated with elevated levels of interleukin-6 (IL-6) and C-reactive protein (CRP) in smokers relative to non-smokers. Smoking is also associated with an increase in oxygen free radical production [42,43,44]. Additionally, smoking causes an increased oxidative stress via several mechanisms including direct damage by radical species and the inflammatory response induced by smoking [45]. Nicotine, which is the main psychoactive compound in cigarettes, stimulates interleukin-6 (IL-6) expression [20,21,22,23,24,25,26,27,28,29,30,31,32,33,34,35,36,37,38,39,40,41,42,43,44,45,46,47]. IL-6 cytokine release during inflammatory events is mainly responsible for CYP3A4 downregulation. Moreover, CYP3A4 is transcriptionally repressed by IL-6 [1,21,46]. These findings suggest that smoking induces IL-6 expression, which, in turn, downregulates CYP3A4, the main enzyme responsible for sildenafil metabolism. Taken together, the aforementioned effects of smoking on CYP3A4 in addition to the effect of smoking on gastric acid secretion and the gastric emptying rate explain the increase in the exposure of sildenafil of 63% in cigarette smokers in the current study. Therefore, the pharmacokinetic alteration of sildenafil exposure in cigarette smokers was probably due to the effect of smoking on the hepatic metabolism of sildenafil, which is mediated by liver cytochrome enzyme (CYP3A4).

In one study, sildenafil decreased both systolic and diastolic blood pressure by 6.0 mmHg and 4.5 mmHg, respectively. The blood pressure-lowering effect of sildenafil did not differ significantly between the normotensive and hypertensive subjects [47]. Similarly, in the current study, sildenafil decreased systolic blood pressure by 4.0 mmHg, 1.0 mmHg and 8.0 mmHg in addition to a reduction in diastolic blood pressure of 4.0 mmHg, 3.0 mmHg and 5.0 mmHg in non-smokers, cigarette smokers and cannabis smokers, respectively. However, the slight blood pressure reductions were comparable among the three groups of the current study with no clinical significance and all subjects tolerated sildenafil.

Although mixed reports regarding the effects of sildenafil on cardiovascular events in patients can be found in the literature, an ECG-12 assessment was unnecessary in the current study because only healthy subjects were included as well as because the study used a single low-dose design. Moreover, most reported incidences of cardiovascular events with sildenafil administration are mainly reported for patients rather than healthy subjects. It is worth mentioning that sildenafil was well tolerated in patients with cardiovascular system disorders [48,49].

Although the interaction is not clinically relevant in healthy subjects, further study of the effects of smoking on the exposure of higher doses of sildenafil in patients with comorbid conditions and multiple-dose regimens is required. In our opinion, a starting dose of 25 mg seems more efficient based on a risk-benefit assessment. Given the increase in the exposure of sildenafil, a similar clinical effect to the lower exposure experienced by non-smokers, the decrease in the TAC and the increased risk of the appearance of comorbid conditions in smokers, a risk-benefit assessment favors an initial lower dose of sildenafil [50]. The suggested lower dose (25 mg) is expected to possess the clinical benefits of the higher dose with a lower risk in smokers, which is associated with a reduction in the overall unnecessary exposure to sildenafil in the population. Therefore, we suggest a starting dose of 25 mg of sildenafil rather than 50 mg in cigarette smokers with a further increase of the dose if clinical effectiveness is not achieved.

## 5. Conclusions

Cigarette smoking increased the bioavailability of sildenafil without altering its pharmacodynamics, safety or tolerability profiles.

## 6. Limitations

In this study, uncontrolled amounts of cannabis delivery by means of smoking would account for the fact that a non-significant interaction between cannabis and sildenafil was shown. In addition, a study of the genetic polymorphisms of the CYP3A4 gene was not performed, which could contribute to the inter-individual variations in metabolism.

## Figures and Tables

**Table 1:** Demographic data of subjects in each study group.

Demographic Data	Group 1	Group 2	Group 3	*p*-Value
Age (year)	30.00 ± 11.04	28.75 ± 8.69	30.42 ± 7.06	0.89
Height (cm)	172.17 ± 6.32	174.08 ± 6.89	172.00 ± 5.61	0.67
Weight (kg)	77.33 ± 11.09	71.67± 9.59	71.17 ± 14.26	0.36
BMI	26.04 ± 3.12	23.21 ± 3.40	22.57 ± 4.22	0.16

Data expressed as mean ± SD; Group 1: non-smokers; Group 2: cigarette smokers; Group 3: cannabis smokers; BMI: body mass index; *p* > 0.05: non-significant.

**Table 2:** Intra-day accuracy validation data for the method of quantitation of sildenafil in human plasma samples by liquid chromatography-tandem mass spectrometry.

	Concentration (ng/mL) of Individual Samples					Intra-Day Accuracy			
Quality Control Sample (QC) *	1	2	3	4	5	Mean	SD	Accuracy %	Accepted QC Samples **
LLOQ (1 ng/mL)	1.01	0.91	1.06	1.02	0.91	0.98	0.07	98.75	√
QCA (3 ng/mL)	2.62	2.60	2.66	2.66	2.64	2.64	0.02	88.04	√
QCB (200 ng/mL)	191.76	201.57	201.56	194.56	197.75	197.44	4.32	98.72	√
QCC (400 ng/mL)	393.90	405.21	437.47	396.13	399.54	406.45	17.86	101.61	√

* Five preparations for the same QC at each concentration level; LLOQ: lower limit of quantitation; QCA: low-concentration QC sample; QCB: medium (mid)-concentration QC sample; QCC: high-concentration QC sample. ** Acceptable validation limit range for accuracy % as per FDA guidance for bioanalytical method validation (√); 80–120% for LLOQ and 85–115% for the low-, mid- and high-concentration QC samples.

**Table 3:** Pharmacokinetic (PK) parameters of sildenafil after administering a single oral dose of sildenafil 50 mg tablets in all study groups.

PK Parameters *	Group 1 (Non-Smokers)	Group 2 (Cigarette Smokers)	Group 3 (Cannabis Smokers)	*p*-Value
C_max_ (ng/mL)	216.85 ± 82	352.11 ± 88	264.306 ± 75	0.001 **
T_max_ (h)	1.25 (0.5–2)	0.75 (0.3–1.5)	0.75 (0.5–4)	-
T1/2 (h)	3.5 (3.0–4.2)	4.2 (3.0–5.6)	4.3(3.2–8.6)	-
AUC_0–t_ (ng·h/mL)	717.40 ± 311	1155.89 ± 542.26	967.29 ± 262	0.033 **
AUC_0–inf_ (ng·h/mL)	726.80 ± 313	1207.10 ± 596.73	1008.11 ± 278	0.029 **

* PK parameters are expressed as the mean ± standard deviation; T_max_ and T1/2 are expressed as the median (range). ** Statistically significant (*p* < 0.05).

**Table 4:** Malondialdehyde plasma concentration and total antioxidant capacity of all study groups.

Oxidative Stress Parameters *	Group 1 (Non-Smokers)	Group 2 (Cigarette Smokers)	Group 3 (Cannabis Smokers)	*p*-Value
MDA (nM/mL)	55 ± 16	66.2 ± 34.6	58 ± 17	*p* > 0.05
TAC (nM/mL)	1.82 ± 0.07	1.36 ± 0.09	1.41 ± 0.09	*p* < 0.05 **

All data are expressed as mean ± SD. MDA: malondialdehyde level in plasma; TAC: total antioxidant capacity. * Surrogate biomarkers; ** statistically significant *p* < 0.05.

## Supplementary Materials

The following are available online at https://www.mdpi.com/article/10.3390/pharmaceutics13060876/s1, Bioanalytical Supplementary File, Pharmacokinetic Supplementary File, Statistical Analysis Supplementary File, Supplementary File.
