# Supplementary material for: Changes in the Pharmacokinetics and Pharmacodynamics of Sildenafil in Cigarette and Cannabis Smokers
Source: Pharmaceutics. 2021 Jun 13;13(6):876. doi: 10.3390/pharmaceutics13060876 (PMC8231986; doi:10.3390/pharmaceutics13060876)
Supplement: Supplementary file 1 [file pharmaceutics-13-00876-s001.zip › pharmaceutics-1235675-sup.pdf]

pharmaceutics

Supplementary Materials: Changes in the Pharmacokinetics and Pharmacodynamics of Sildenafil in Cigarette and Cannabis Smokers

Mohammed Murtadha, Mohamed Ahmed Raslan, Sarah Farid Fahmy and Nagwa Ali Sabri

1. Bioanalytical Supplementary File

Bioanalytical method

This analysis was conducted in conformity with the study protocol and FDA Guidance for Analytical Methods Validation. The individual and mean data for the pre-study validation, recovery and stability tests results are expressed using three significant figures.

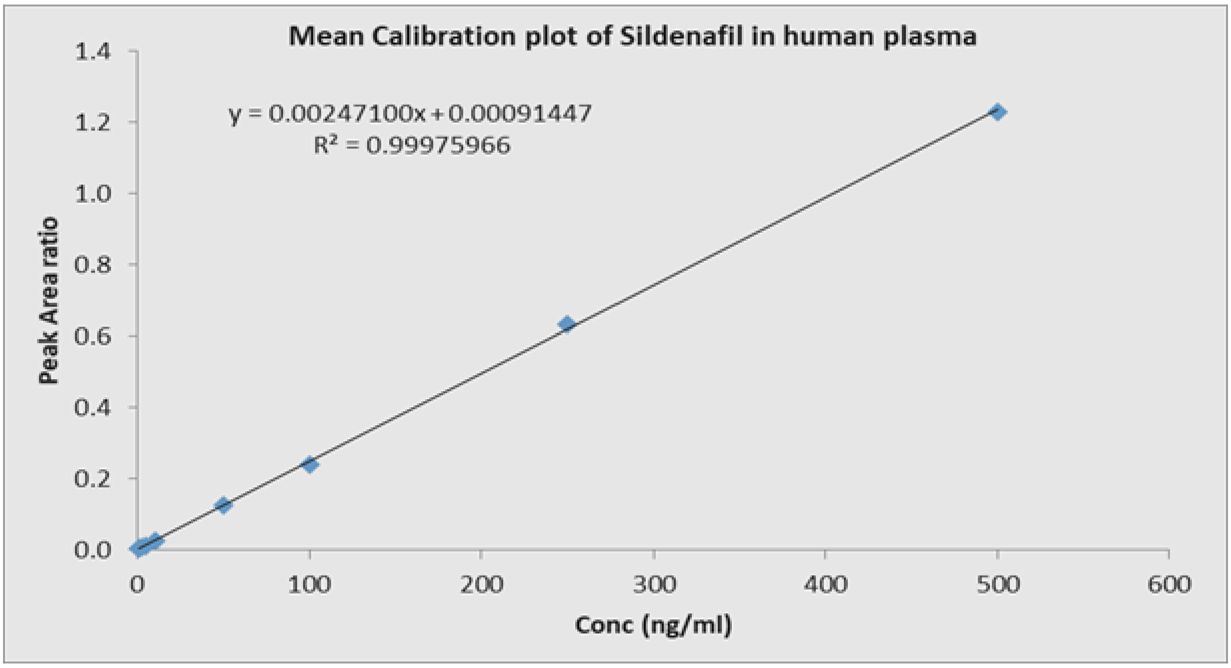

Figure S1. Mean Calibration Plot of Sildenafil in Human plasma.

Quality control samples concentrations were finally defined as presented in the following table:

Table S1. : Quality Control Samples Definition for Sildenfil**.**

**QC Type**	**QC code**	**Range definition**	**Sildenafil Defined value Conc. (ng/ml)**
Low QC sample	QCA	3 × LLOQ	3
Mediu m QC sample	QCB	Average between low and high QC	200
High QC sample	QCC	75–90% of highest calibration	400

The intra- and inter-day precision and accuracy validation tests were carried out using a batch containing the following samples:

- A calibration curve in triplicate containing standards as defined above for each validation batch;

- LLOQ and three quality control (QC) samples of each of the QC types defined above;

- The quality control (QC) samples were prepared independently, i.e. from different master solutions, from the standards samples.

Table S2. Inter-day Validation Data of Sildenafil.

Sample Code	Concentration (ng/ml) of individual samples									Inter days Accuracy		
	Day 1			Day 2			Day 3			Mean	SD	Accuracy %
	Rep1	Rep2	Rep3	Rep1	Rep2	Rep3	Rep1	Rep2	Rep3			
LLOQ (1ng/ml)	1.004	0.989	0.906	0.997	0.950	0.945	1.035	1.020	0.965	0.979	0.041	97.886
QCA (3ng/ml)	2.842	2.712	2.719	2.828	2.840	2.651	2.824	2.670	2.861	2.772	0.083	92.391
QCB (200ng/ml)	193.222	198.625	201.006	196.018	204.223	202.887	190.445	196.728	198.339	197.944	4.438	98.972
QCC (400ng/ml)	396.852	403.002	434.300	404.413	408.639	435.922	388.302	399.618	431.992	411.449	17.891	102.862

Accuracy Limits: 85–115%, and 80–120% for LLOQ

Table S3. Analytical Precision for the Analysis of Sildenafil performed on three sets of Standard Curves on the same day.

Spiked Concentration s(ng/ml)	Peak Area Ratio			Mean	SD	CV%
	1	2	3			
1	0.00320	0.00363	0.00334	0.00339	0.00022	6.420
2.5	0.00778	0.00785	0.00773	0.00779	0.00006	0.790
5	0.01273	0.01234	0.01326	0.01278	0.00046	3.597
10	0.02444	0.02420	0.02455	0.02440	0.00018	0.740
50	0.12417	0.12661	0.12536	0.12538	0.00122	0.974
100	0.23949	0.24038	0.23763	0.23917	0.00141	0.588
250	0.64516	0.63153	0.62281	0.63317	0.01126	1.778
500	1.25239	1.22974	1.21046	1.23087	0.02099	1.705
Slope	0.00252	0.00247	0.00243	0.00247		
R^2	0.99968	0.99980	0.99978	0.99975		
Intercept	−0.00026	0.00121	0.00180			

Precision Limits: CV% <15%

Table S4. Accuracy determination of Sildenafil in Plasma.

Spiked Concentration (ng/ml)	Recovery			Mean	SD	Recovery %
	1	2	3			
1	0.9256	1.0977	0.9797	1.0010	0.0880	100.101
2.5	2.7771	2.8082	2.7589	2.7814	0.0249	111.255
5	4.7804	4.6255	4.9958	4.8005	0.1860	96.011
10	9.5191	9.4229	9.5662	9.5027	0.0730	95.027
50	49.8804	50.8688	50.3629	50.3707	0.4942	100.741
100	96.5520	96.9110	95.7955	96.4195	0.5694	96.420
250	260.7207	255.2078	251.6789	255.8692	4.5570	102.348
500	506.4661	497.3009	489.4979	497.7550	8.4932	99.551

Average Recovery % = 100.182 %, Accuracy Limits: 85–115%, except for LLOQ: 80–120%

Table S5. Intermediate precision Data for Sildenafil in Plasma performed on three sets of Quality controls on three different days.

Sample Code	Peak area ratios of individual samples(a)									Intermediate Precision	
	Day 1			Day 2			Day 3			Mean	SD
	Rep1	Rep2	Rep3	Rep1	Rep2	Rep3	Rep1	Rep2	Rep3		
LLOQ (1ng/ml)	0.00340	0.00336	0.00315	0.00338	0.00326	0.00325	0.00347	0.00343	0.00330	0.0033	0.0001
QCA (3ng/ml)	0.00794	0.00762	0.00763	0.00790	0.00793	0.00746	0.00789	0.00751	0.00798	0.0078	0.0002
QCB (200ng/ml)	0.47836	0.49172	0.49760	0.48528	0.50555	0.50225	0.47150	0.48703	0.49101	0.4900	0.0110
QCC (400ng/ml)	0.98153	0.99673	1.07407	1.00022	1.01066	1.07808	0.96041	0.98837	1.06837	1.0176	0.0442

Table S6. Recovery and Matrix effect (Sildenafil): For evaluation of matrix effect, extracting two sets of samples; one set contains the analyte added to an extracted matrix (post-extraction sample), and the other contains the analyte in mobile phase, solvent, or buffer (external solution). Both sets of samples are prepared with equivalent concentrations of the analyte and then are processed identically

Matrix Effect % = (B/A) × 100

Recovery % = (C/B) × 100

Where, A = External solution Peak Area, B = Post extraction Peak Area, C = Extracted matrix Peak Area.

Internal standard					
QC low (3ng/ml)					
Lot No	Peak Area			Matrix effect	Recovery
	Spiked plasma	Post extraction	External		
1	1586891	1551736	1424745	105.030	106.382
2	1361183	1411578	1492555	95.544	91.251
3	1342204	1520679	1506042	102.928	89.979
4	1559356	1473077	1422294	99.706	104.536
5	1431202	1488320	1556408	100.738	95.945
6	1445175	1504747	1462462	101.850	96.882
Mean	1454335	1491690	1477418	100.966	97.496
SD	100469.113	47732.804	51622.988	3.231	6.735
CV%	6.908	3.200	3.494	3.200	6.908
QC Med (200ng/ml)					
Lot No	Peak Area			Matrix effect	Recovery
	Spiked plasma	Post extraction	External		
1	1408386	1493933	1507415	99.681	93.468
2	1518842	1580223	1472986	105.438	100.799
3	1654679	1564870	1550939	104.414	109.813
4	1615931	1474129	1442044	98.359	107.242
5	1646100	1433322	1509415	95.636	109.244
6	1567295	1494380	1509520	99.710	104.014
Mean	1568539	1506810	1498720	100.540	104.097
SD	93597.410	55766.097	37169.498	3.721	6.212
CV%	5.967	3.701	2.480	3.701	5.967
QC High (400ng/ml)					
Lot No	Peak Area			Matrix effect	Recovery

	Spiked plasma	Post extraction	External		
1	1604244	1559750	1544091	104.278	105.941
2	1598166	1465211	1454753	97.958	105.539
3	1616352	1548456	1489962	103.523	106.740
4	1550869	1563028	1559568	104.498	102.416
5	1563325	1438427	1444818	96.167	103.238
6	1537571	1510849	1481344	101.009	101.538
Mean	1578421	1514287	1495756	101.239	104.235
SD	32097.242	52507.339	46744.937	3.510	2.120
CV%	2.034	3.467	3.125	3.467	2.034

2. Pharmacokinetic Supplementary File

Pharmacokinetic Data:

Volunteers are divided to three groups

Group1 = Non smokers , Group2 = Cigarette smokers , Group3 = THC smokers

Table S7. Pharmacokinetic parameters of Sildenafil following administration of single oral dose of Sildenafil 50 mg Tablet "Viagra 50mg Tablet" to 12 (Non-smokers) volunteers.

Subject	T_{max}	C_{max}	AUC_{0-t}	AUC_{0-inf}	K_{el}	$T_{1/2}$	MRT_{inf}
1	2.000	179.003	553.737	559.762	0.212	3.262	4.426
2	0.500	312.320	750.860	756.928	0.207	3.349	3.468
3	0.750	236.411	601.524	607.440	0.201	3.443	4.020
4	1.000	260.442	604.744	615.850	0.186	3.717	3.961
5	0.750	305.150	915.693	926.545	0.198	3.502	4.458
6	1.500	228.077	959.385	968.492	0.211	3.283	4.574
7	1.000	266.875	911.647	943.156	0.162	4.289	5.326
8	0.750	262.742	528.777	534.675	0.187	3.703	3.503
9	1.500	71.658	298.955	304.480	0.199	3.478	4.358
10	1.500	269.745	1455.093	1462.766	0.228	3.037	4.870
11	2.000	121.043	656.218	662.660	0.195	3.555	5.301
12	1.500	88.778	372.313	378.529	0.187	3.701	4.258
Mean	**1.229**	**216.854**	**717.412**	**726.773**	**0.198**	**3.527**	**4.377**
SD	**0.505**	**82.474**	**311.496**	**313.747**	**0.017**	**0.315**	**0.599**
CV%	**41.100**	**38.032**	**43.419**	**43.170**	**8.463**	**8.927**	**13.692**

Table S8. Pharmacokinetic parameters of Sildenafil following administration of single oral dose of Sildenafil 50mg Tablet "Viagra 50mg Tablet" to 12 (Cigarette smokers) volunteers.

Subject	T_{max}	C_{max}	AUC_{0-t}	AUC_{0-inf}	K_{el}	$T_{1/2}$	MRT_{inf}
1	1.000	457.020	1336.566	1418.450	0.144	4.808	6.302
2	0.750	496.187	2520.547	2742.941	0.122	5.666	8.096
3	1.000	191.331	562.733	594.158	0.149	4.655	5.810
4	0.333	360.094	965.776	1002.734	0.165	4.202	5.010
5	1.500	300.862	1566.218	1692.319	0.133	5.214	7.968
6	0.500	379.622	1070.714	1084.339	0.206	3.363	3.478
7	0.333	361.301	537.402	555.483	0.165	4.210	3.950
8	1.500	431.257	1501.268	1505.852	0.231	2.995	3.737
9	0.500	335.471	933.950	939.938	0.202	3.433	3.332
10	1.000	331.974	1183.945	1191.042	0.213	3.247	4.691
11	0.500	362.607	972.436	1029.499	0.136	5.113	5.935
12	0.750	217.596	719.375	729.001	0.204	3.399	4.211
Mean	**0.806**	**352.110**	**1155.911**	**1207.146**	**0.173**	**4.192**	**5.210**
SD	**0.406**	**88.573**	**542.268**	**596.738**	**0.037**	**0.898**	**1.641**
CV%	**50.370**	**25.155**	**46.913**	**49.434**	**21.442**	**21.427**	**31.491**

Table S9. Pharmacokinetic parameters of Sildenafil following administration of single oral dose of Sildenafil 50mg Tablet "Viagra 50mg Tablet" to 12 (THC smokers) volunteers.

Subject	T_{max}	C_{max}	AUC_{0-t}	AUC_{0-inf}	K_{el}	$T_{1/2}$	MRT_{inf}
1	0.750	243.584	705.603	716.653	0.161	4.293	4.016
2	1.000	224.109	677.223	698.980	0.137	5.052	4.975
3	0.500	347.884	1221.926	1275.299	0.161	4.296	5.300
4	3.500	164.418	1099.042	1127.613	0.160	4.330	7.058
5	1.500	361.525	1344.051	1424.975	0.150	4.613	6.989
6	4.000	165.348	774.269	884.949	0.080	8.681	10.265
7	0.750	317.421	926.428	949.664	0.180	3.856	4.722
8	1.500	194.552	707.070	716.850	0.211	3.288	4.054
9	0.500	392.509	972.803	990.914	0.183	3.794	3.926
10	0.750	237.032	1406.440	1477.866	0.143	4.858	7.175
11	0.500	265.697	703.524	724.074	0.166	4.168	4.463
12	0.750	257.596	1069.116	1109.596	0.158	4.395	5.947
Mean	**1.333**	**264.306**	**967.291**	**1008.119**	**0.158**	**4.635**	**5.741**
SD	**1.184**	**75.778**	**262.199**	**278.847**	**0.031**	**1.359**	**1.869**
CV%	**88.789**	**28.671**	**27.106**	**27.660**	**19.897**	**29.327**	**32.561**

Table S10. Assessment of Sildenafil absorption rate C_{max}/ $AUC_{0–t}$ (h^{-1}).   .

Subject	THC smokers	Cigarette smokers	Non-smokers
1	0.345	0.342	0.323
2	0.331	0.197	0.416
3	0.285	0.340	0.393
4	0.150	0.373	0.431
5	0.269	0.192	0.333
6	0.214	0.355	0.238
7	0.343	0.672	0.293
8	0.275	0.287	0.497
9	0.403	0.359	0.240
10	0.169	0.280	0.185
11	0.378	0.373	0.184
12	0.241	0.302	0.238
Mean	**0.283**	**0.339**	**0.314**
SD	**0.080**	**0.122**	**0.102**

Table S11. Percent of the area measured by $AUC_{0–t}$ relative to the extrapolated total $AUC_{0–inf}$ of Sildenafil.

Subject	*THC smokers*	*Cigarette smokers*	*Non-smokers*
1	98.458	94.227	98.924
2	96.887	91.892	99.198
3	95.815	94.711	99.026
4	97.466	96.314	98.197
5	94.321	92.549	98.829
6	87.493	98.743	99.060
7	97.553	96.745	96.659
8	98.636	99.696	98.897
9	98.172	99.363	98.186
10	95.167	99.404	99.475
11	97.162	94.457	99.028
12	96.352	98.679	98.358
Mean	**96.124**	**96.398**	**98.653**
SD	**3.019**	**2.796**	**0.743**

Table S12. Plasma concentration levels of Sildenafil 50mg Tablet after administration of "Viagra 50mg Tablet" to 12 (Non-Smokers) volunteers.

Volunteer's Code	Blood Sampling Intervals (hour)															
	**0**	**0.167**	**0.333**	**0.5**	**0.75**	**1**	**1.5**	**2**	**2.5**	**3**	**3.5**	**4**	**6**	**8**	**10**	**12**	**24**
**25**	0.000	1.351	1.493	2.367	16.382	91.348	93.617	179.003	141.840	119.512	80.124	62.864	25.576	12.526	6.049	4.687	1.280
**26**	0.000	41.933	269.588	312.320	275.634	237.173	181.500	135.199	94.521	61.142	45.109	46.592	32.879	13.433	7.349	6.313	1.256
**27**	0.000	2.311	71.539	190.319	236.411	185.046	146.951	102.447	80.470	69.268	46.224	36.371	29.129	11.259	7.020	7.278	1.191
**28**	0.000	1.974	19.296	199.947	217.400	260.442	156.675	110.735	99.436	65.320	50.081	43.651	16.764	8.063	7.103	5.075	2.071
**29**	0.000	2.438	33.533	137.797	305.150	293.297	205.749	142.379	134.823	115.127	88.606	78.467	42.807	22.377	13.779	11.780	2.148
**30**	0.000	2.057	19.878	140.741	200.990	202.549	228.077	196.701	156.415	133.136	120.201	101.382	34.760	20.275	18.635	13.902	1.923
**31**	0.000	10.378	54.595	161.240	229.937	266.875	249.001	181.811	126.725	102.407	82.791	65.970	38.003	20.717	15.203	11.060	5.092
**32**	0.000	3.576	4.409	162.644	262.742	256.740	178.272	104.509	66.057	37.739	26.100	20.946	13.360	8.971	6.670	4.918	1.104
**33**	0.000	15.517	42.703	52.955	55.538	70.274	71.658	66.753	54.521	43.204	38.505	26.618	14.012	4.631	3.804	2.270	1.101
**34**	0.000	1.998	92.609	139.521	229.651	247.729	269.745	258.132	242.227	200.632	190.702	148.715	71.723	52.714	38.710	22.361	1.751
**35**	0.000	1.827	23.983	58.490	90.259	113.009	116.016	121.043	89.802	81.762	70.152	62.672	44.979	23.293	17.473	11.147	1.256
**36**	0.000	20.304	54.896	62.789	67.099	87.757	88.778	83.904	67.344	54.198	47.941	33.471	18.107	6.021	5.258	2.620	1.164
**Mean**	**0.000**	**8.805**	**57.377**	**135.094**	**182.266**	**192.687**	**165.503**	**140.218**	**112.848**	**90.287**	**73.878**	**60.643**	**31.842**	**17.023**	**12.255**	**8.618**	**1.778**
**SD**	**0.000**	**12.173**	**72.094**	**82.916**	**97.531**	**80.959**	**65.035**	**54.660**	**52.145**	**46.610**	**45.235**	**36.079**	**16.660**	**12.976**	**9.756**	**5.754**	**1.114**
**CV%**	**0.000**	**138.249**	**125.650**	**61.377**	**53.510**	**42.016**	**39.295**	**38.982**	**46.208**	**51.625**	**61.229**	**59.494**	**52.320**	**76.224**	**79.609**	**66.774**	**62.654**

Table S13. Plasma concentration levels of Sildenafil 50mg Tablet after administration of "Viagra 50mg Tablet" to 12 (Cigarettes Smokers) volunteers.

Volunteer's Code	Blood Sampling Intervals (hour)															
	**0**	**0.167**	**0.333**	**0.5**	**0.75**	**1**	**1.5**	**2**	**2.5**	**3**	**3.5**	**4**	**6**	**8**	**10**	**12**	**24**
**13**	0.000	1.037	1.568	281.352	340.614	457.020	320.992	245.903	211.085	159.631	118.142	95.494	45.561	30.818	18.358	16.198	11.805
**14**	0.000	1.025	194.433	476.519	496.187	472.300	457.746	413.349	377.488	251.471	192.019	175.810	119.489	79.849	61.512	48.107	27.207
**15**	0.000	1.250	13.835	154.492	161.723	191.331	148.564	120.778	75.412	71.712	45.339	34.581	19.407	11.993	6.790	5.321	4.679
**16**	0.000	54.350	360.094	276.130	271.235	245.859	186.355	147.091	123.904	110.708	105.008	98.111	37.662	18.489	10.515	7.158	6.097
**17**	0.000	1.098	20.394	140.564	224.469	221.710	300.862	290.923	253.583	208.623	145.168	138.921	71.836	47.769	34.734	30.225	16.765
**18**	0.000	93.307	370.407	379.622	331.198	315.823	257.007	237.215	178.471	135.593	89.129	76.307	26.758	16.107	9.895	6.666	2.808
**19**	0.000	18.249	361.301	358.005	263.176	178.339	112.486	63.316	49.350	41.557	35.540	26.390	11.462	5.393	4.187	3.262	2.977
**20**	0.000	12.721	231.004	248.017	281.967	386.361	431.257	342.327	283.272	207.990	129.377	106.706	72.326	41.125	16.958	13.703	1.061
**21**	0.000	116.488	217.700	335.471	318.350	257.389	243.815	229.392	152.293	93.067	73.798	48.191	33.629	14.181	10.187	7.512	1.209
**22**	0.000	6.593	9.619	78.547	248.900	331.974	248.543	227.729	188.912	153.379	126.540	113.069	46.113	36.197	25.138	20.040	1.515
**23**	0.000	106.432	303.886	362.607	331.083	278.197	188.091	137.112	119.057	90.851	69.854	60.249	33.895	18.825	13.612	12.078	7.736
**24**	0.000	1.373	59.740	108.716	217.596	200.966	168.415	160.896	135.785	101.357	67.870	58.866	28.332	14.732	9.242	7.091	1.963
**Mean**	**0.000**	**34.493**	**178.665**	**266.670**	**290.542**	**294.772**	**255.344**	**218.003**	**179.051**	**135.495**	**99.815**	**86.058**	**45.539**	**27.957**	**18.427**	**14.780**	**7.152**
**SD**	**0.000**	**45.552**	**150.614**	**123.786**	**84.249**	**100.234**	**107.503**	**99.325**	**92.299**	**62.829**	**45.043**	**44.092**	**29.711**	**20.839**	**16.007**	**12.952**	**7.927**
**CV%**	**0.000**	**132.059**	**84.300**	**46.419**	**28.997**	**34.004**	**42.101**	**45.561**	**51.549**	**46.370**	**45.126**	**51.235**	**65.244**	**74.541**	**86.865**	**87.633**	**110.842**

Table S14. Plasma concentration levels of Sildenafil 50mg Tablet after administration of "Viagra 50mg Tablet" to 12 (THC Smokers) volunteers.

Volunteer's Code	Blood Sampling Intervals (hour)																
	**0**	**0.167**	**0.333**	**0.5**	**0.75**	**1**	**1.5**	**2**	**2.5**	**3**	**3.5**	**4**	**6**	**8**	**10**	**12**	**24**
**1**	0.000	5.012	71.369	162.582	243.584	202.097	189.114	134.994	96.115	95.259	86.496	65.937	22.487	13.308	9.537	5.361	1.784
**2**	0.000	1.175	1.897	12.244	114.190	224.109	215.196	153.936	112.689	89.099	69.335	66.840	27.136	13.212	8.549	6.027	2.985
**3**	0.000	15.268	246.040	347.884	345.476	316.980	293.115	224.096	187.840	156.406	136.006	100.573	37.080	21.830	13.905	11.272	8.611
**4**	0.000	1.161	1.237	1.418	1.762	2.031	61.980	119.720	161.102	161.883	164.418	162.428	87.178	59.115	35.463	24.086	4.574
**5**	0.000	1.258	1.441	1.888	2.919	248.697	361.525	288.213	238.373	191.280	152.572	130.240	61.319	43.786	28.053	18.737	12.160
**6**	0.000	1.192	1.389	1.699	2.393	5.284	46.097	66.856	111.167	116.362	145.747	165.348	59.473	22.810	12.769	11.140	8.837
**7**	0.000	1.106	1.596	192.737	317.421	290.095	199.666	156.230	128.349	121.032	104.198	100.803	33.969	21.154	13.074	7.922	4.177
**8**	0.000	25.634	72.675	92.590	156.833	184.608	194.552	146.285	115.715	113.411	89.822	71.157	29.663	12.673	7.208	4.666	2.062
**9**	0.000	77.506	306.034	392.509	360.370	301.546	215.616	186.830	127.898	100.569	75.056	58.887	31.921	18.289	11.583	7.700	3.309
**10**	0.000	3.423	4.845	137.110	237.032	205.559	179.749	170.465	168.484	166.876	165.053	154.894	92.662	61.309	40.500	24.444	10.192
**11**	0.000	39.712	206.909	265.697	256.755	224.558	157.849	122.258	81.839	64.987	59.416	51.151	23.692	13.217	8.117	5.857	3.417
**12**	0.000	1.268	79.276	127.310	257.596	228.511	218.006	187.722	145.733	122.537	107.632	98.666	56.185	32.048	22.024	15.157	6.385
**Mean**	**0.000**	**14.476**	**82.892**	**144.639**	**191.361**	**202.840**	**194.372**	**163.134**	**139.609**	**124.975**	**112.979**	**102.244**	**46.897**	**27.729**	**17.565**	**11.864**	**5.708**
**SD**	**0.000**	**23.365**	**109.231**	**135.497**	**133.484**	**101.696**	**85.414**	**56.096**	**43.705**	**37.045**	**38.239**	**41.785**	**24.269**	**17.675**	**11.325**	**7.159**	**3.453**
**CV%**	**0.000**	**161.401**	**131.774**	**93.680**	**69.755**	**50.136**	**43.943**	**34.386**	**31.305**	**29.642**	**33.846**	**40.868**	**51.750**	**63.741**	**64.475**	**60.341**	**60.495**

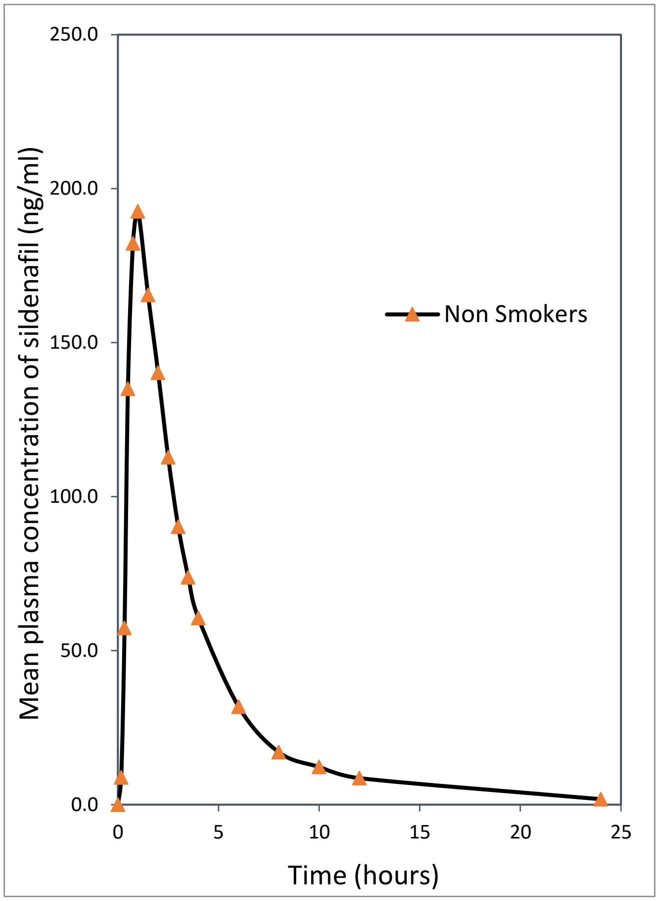

Figure S2. Mean sildenafil plasma concentration versus time curves after oral administration of a single dose sildenafil 50 mg in Non-smoker group.

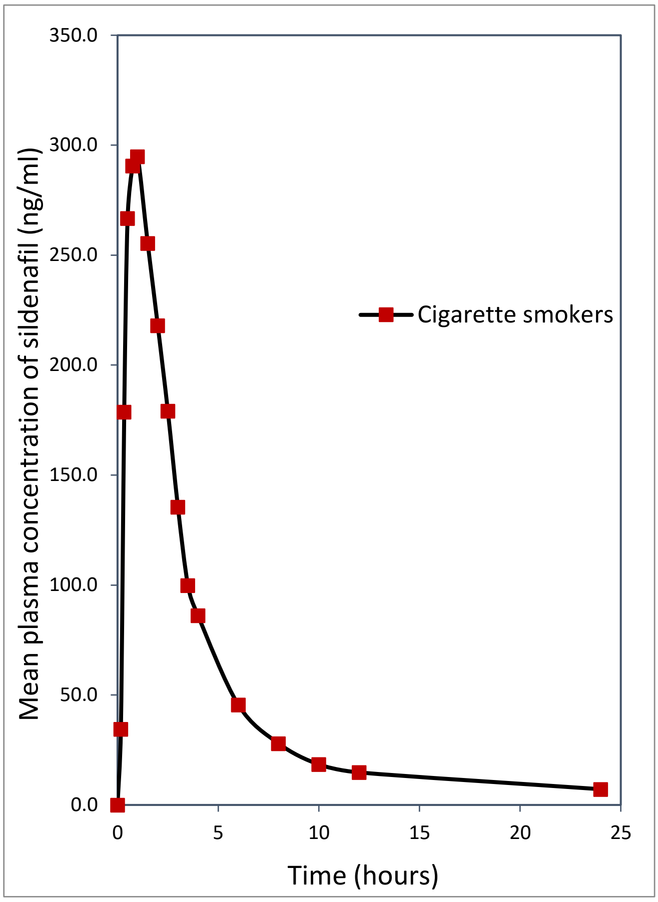

Figure S3. Mean sildenafil plasma concentration versus time curves after oral administration of a single dose sildenafil 50 mg in Cigarette-smoker group.

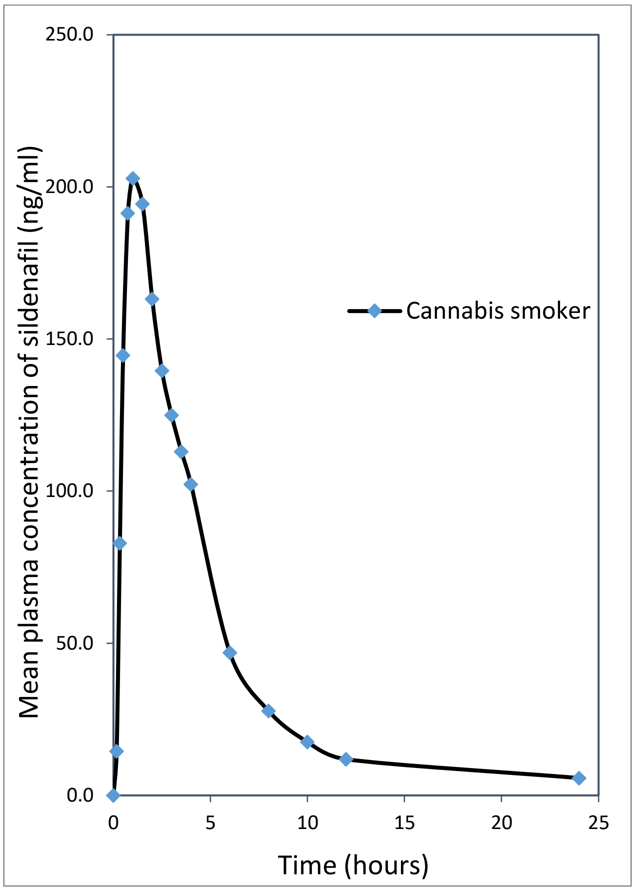

Figure S4. Mean sildenafil plasma concentration versus time curves after oral administration of a single dose sildenafil 50 mg in Cannabis-smoker group.

3. Statistical Analysis Supplementary File

ANOVA Table for C_{max}

Completely Randomized ANOVA for Cmax

Source	DF	SS	MS	F	P
Group	2	113022	56511.2	8.31	**0.0012**
Error	33	224284	6796.5		
Total	35	337306			

Grand Mean 277.76 CV 29.68

Group	Mean
1(THC Smoker)	264.31
2(cig Smoker)	352.11
3(Non-smoker)	216.85

Observations per Mean 12

Standard Error of a Mean 23.799

Std Error (Diff of 2 Means) 33.656

LSD All-Pairwise Comparisons Test of Cmax by Group

Group	Mean	Homogeneous Groups
2(cig Smoker)	352.11	A
1(THC Smoker)	264.31	B
3(Non-smoker)	216.85	B

Alpha 0.05 Standard Error for Comparison 33.656

Critical T Value 2.035 Critical Value for Comparison 68.474

There is a significant difference between **[2(cig Smoker)]**and**[3(Non-smoker)]**

There is a significant difference between **[2(cig Smoker)]**and**[1(THC Smoker)]**

There are no significant difference between **[3(Non-smoker)]**and**[1(THC Smoker)]**

ANOVA Table for T_{max}

Completely Randomized ANOVA for Tmax

Source	DF	SS	MS	F	P
Group	2	1.8758	0.93790	1.54	**0.2284**
Error	33	20.0348	0.60711		
Total	35	21.9106			

Grand Mean 1.1227 CV 69.40

Group	Mean
1(THC Smoker)	1.3333
2(cig Smoker)	0.8055
3(Non-smoker)	1.2292

Observations per Mean 12

Standard Error of a Mean 0.2249

Std Error (Diff of 2 Means) 0.3181

LSD All-Pairwise Comparisons Test of Tmax by Group

Group	Mean	Homogeneous Groups
1(THC Smoker)	1.3333	A
3(Non-smoker)	1.2292	A
2(cig Smoker)	0.8055	A

Alpha	0.05	Standard Error for Comparison	0.3181
Critical T Value	2.035	Critical Value for Comparison	0.6472

There are no significant pairwise differences among the means.

ANOVA Table for AUC_{0-t}

Completely Randomized ANOVA for AUC_{0-t}

**Source**	**DF**	**SS**	**MS**	**F**	**P**
Group	2	1161192	580596	3.79	**0.0330**
Error	33	5058152	153277		
Total	35	6219343			

Grand Mean 946.87 CV 41.35

**Group**	**Mean**
1(THC Smoker)	967.3
2(cig Smoker)	1155.9
3(Non-smoker)	717.4

Observations per Mean 12
Standard Error of a Mean 113.02
Std Error (Diff of 2 Means) 159.83

LSD All-Pairwise Comparisons Test of AUC_{0-t} by Group

**Group**	**Mean**	**Homogeneous Groups**
2(cig Smoker)	1155.9	A
1(THC Smoker)	967.29	AB
3(Non-smoker)	717.41	B

Alpha	0.05	Standard Error for Comparison	159.83
Critical T Value	2.035	Critical Value for Comparison	325.18

There is a significant difference between **[2(cig Smoker)]**and**[3(Non-smoker)]**
There are no significant difference between **[1(THC Smoker)]**and**[2(cig Smoker)]**
There are no significant difference between **[1(THC Smoker)]**and**[3(Non-smoker)]**

ANOVA Table for $AUC_{0–inf}$

Completely Randomized AOV for $AUC_{0–inf}$

Source	DF	SS	MS	F	P
Group	2	1398101	699050	3.94	**0.0292**
Error	33	5855182	177430		
Total	35	7253283			

Grand Mean 980.68 CV 42.95

Group	Mean
1(THC Smoker)	1008.1
2(cig Smoker)	1207.1
3(Non-smoker)	726.8

Observations per Mean 12
Standard Error of a Mean 121.60
Std Error (Diff of 2 Means) 171.96

LSD All-Pairwise Comparisons Test of AUC_{0-inf} by Group

Group	Mean	Homogeneous Groups
2(cig Smoker)	1207.1	A
1(THC Smoker)	1008.1	AB
3(Non-smoker)	726.77	B

Alpha 0.05 Standard Error for Comparison 171.96
Critical T Value 2.035 Critical Value for Comparison 349.86

There is a significant difference between **[2(cig Smoker)]**and**[3(Non-smoker)]**
There are no significant difference between **[1(THC Smoker)]**and**[2(cig Smoker)]**
There are no significant difference between **[1(THC Smoker)]**and**[3(Non-smoker)]**

Table S15. Blood pressure and pulse rate measurements after administartoin of sildenafil 50mg in non-smokers.

	Non-Smokers														
**Subject's Code/Time (hours)**	**Systolic Blood Pressure(mmHg)**					**Diastolic Blood Pressure(mmHg)**					**Pulse Rate (bpm)**				
	[illegible]	**2**	**4**	**6**	**10**	**0**	**2**	**4**	**6**	**10**	**0**	**2**	**4**	**6**	**10**
25	[illegible]	110	120	120	110	80	70	70	70	70	74	70	80	77	72
26	[illegible]	110	110	120	110	80	70	70	80	70	73	79	77	71	80
27	[illegible]	110	120	110	110	80	70	80	70	80	71	77	79	72	73
28	[illegible]	120	110	120	120	80	80	80	70	80	73	79	77	71	72
29	[illegible]	110	120	110	120	80	70	80	80	80	74	77	74	82	83
30	[illegible]	110	110	120	120	70	70	80	70	80	77	82	77	80	82
31	[illegible]	110	120	120	110	70	70	80	80	70	72	74	71	72	77
32	[illegible]	110	120	110	120	80	70	80	70	80	71	78	80	83	77
33	[illegible]	110	120	110	120	70	70	80	70	80	71	75	72	77	74
34	[illegible]	120	110	110	110	70	70	80	70	80	74	80	71	77	80
35	[illegible]	120	110	110	120	75	70	80	70	70	77	79	78	80	83
36	[illegible]	120	110	120	110	80	80	80	70	80	77	71	80	82	79

Table S16. Blood pressure and pulse rate measurements after administartoin of sildenafil 50mg in cigarette smokers.

Subject's Code/Time (hours)	Cigarette Smokers														
	Systolic Blood Pressure(mmHg)					Diastolic Blood Pressure(mmHg)					Pulse Rate (bpm)				
	0	2	4	6	10	0	2	4	6	10	0	2	4	6	10
13	100	100	110	120	110	70	60	70	80	70	84	87	84	81	80
14	100	100	120	110	120	70	60	70	70	70	84	86	82	80	77
15	110	110	110	120	110	70	70	70	80	80	73	76	72	79	76
16	100	100	120	120	110	60	70	70	80	70	72	76	70	74	72
17	120	110	120	110	120	80	70	80	80	80	81	84	78	76	74
18	115	110	120	120	120	70	70	70	70	80	86	88	84	80	75
19	110	120	110	110	120	70	80	70	80	80	73	74	77	79	80
20	120	110	110	120	110	80	70	80	80	70	77	78	71	79	82
21	110	120	110	110	120	80	70	75	75	80	71	75	82	72	71
22	110	110	120	120	110	70	70	80	80	70	71	73	77	79	82
23	110	110	120	120	110	70	70	80	70	80	72	79	71	80	72
24	120	110	125	120	110	70	70	80	80	70	82	80	72	74	77

Table S17. Blood pressure and pulse rate measurements after administartoin of sildenafil 50mg in cannabis smokers.

Subject's Code/Time(hours)	Cannabis Smokers														
	Systolic Blood Pressure(mmHg)					Diastolic Blood Pressure(mmHg)					Pulse Rate (bpm)				
	0	2	4	6	10	0	2	4	6	10	0	2	4	6	10
1	110	100	110	120	110	70	60	70	70	70	75	80	78	76	73
2	120	110	120	120	120	80	70	80	80	70	80	85	80	82	79
3	100	100	120	120	110	70	60	70	80	70	85	88	81	79	76
4	100	100	110	120	120	70	65	65	70	80	77	79	76	71	72
5	100	100	110	110	110	70	70	70	70	70	71	73	73	71	72
6	110	100	110	120	120	70	70	70	70	80	63	70	70	69	68
7	110	100	120	120	110	70	70	75	80	70	81	85	83	80	78
8	110	100	110	120	110	70	70	75	80	70	80	82	76	73	71
9	110	100	110	120	110	70	60	70	70	70	66	69	70	72	74
10	110	100	120	110	120	70	65	70	70	80	74	78	70	71	75
11	110	100	110	110	120	75	70	75	80	80	84	86	81	79	77
12	100	90	110	120	110	70	60	70	80	70	78	80	79	77	80

Table S18. The study diet food details and calories.

Lunch	calories
1/2 Chicken	500
rice (1cup)	130
bread	80
frenchfries	220
tahini	90
Salad	70
total calories	**1090**

Breakfast	calories
Beans sandwich X 2	520
Falafel sandwich	330
total calories	**850**

Adults need genrally a total daily intake of 2000 calorie for women to 2500 calorie for men

Source: https://www.nhs.uk/common-health-questions/food-and-diet/what-should-my-daily-intake-of-calories-be/#:~:text=An%20ideal%20daily%20in-take%20of,women%20and%202%2C500%20for%20men.
